# Successful outcome of a giant inguinoscrotal hernia: a novel two-staged repair using preoperative progressive pneumoperitoneum and transversus abdominis release

**DOI:** 10.1093/jscr/rjaa511

**Published:** 2020-12-17

**Authors:** Derek B Miller, Logan Reed

**Affiliations:** College of Medicine, Florida State University, Tallahassee, FL, USA; College of Medicine, Florida State University, Tallahassee, FL, USA

**Keywords:** giant inguinoscrotal hernia, preoperative progressive pneumoperitoneum, transversus abdominis release

## Abstract

Giant inguinoscrotal hernias, defined as the extension beyond the midpoint of the inner thigh, continue to require multi-step approaches due to their complexity. Although rare in developed countries, they are commonly present in rural areas after years of neglect. This consequently allows the abdomen to maladapt to lower volumes, creating a loss of domain. Here, we present a giant left inguinoscrotal hernia managed with a unique multi-stage approach, aimed to minimize commonly encountered perioperative complications associated with abdominal hypertension. The combined two-staged approach used begins with preoperative progressive pneumoperitoneum, followed by the combined procedures of laparotomy hernia repair (Stoppa technique) and transversus abdominis release, thereby promoting a tension-free closure that is able to accommodate the reduced contents. Various modalities used in treating these hernias have been previously described; however, to our knowledge, the combined use of techniques described here has not been reported.

## INTRODUCTION

Giant inguinoscrotal hernias (GIHs) classically extend below the midpoint of the inner thigh, while upright [1]. Progression of hernias to this size is a considerably long process, generally requiring several years to develop before presenting [2]. Psychosocial factors such as fear, finances, low self-esteem and poor socialization habits may propagate the continuous delay of medical care, consequently further complicating the reconstruction [3]. The reconstruction becomes complex due to the maladaptive loss of abdominal domain which can no longer accommodate herniated contents without the increased risk of abdominal hypertension. Perioperative complications associated with an increased abdominal pressure may incite fatal cardiorespiratory failure by way of cephalad diaphragm displacement and compression of the inferior vena cava [4]. Several surgical modalities are described in the literature with varying combinations in treating GIHs [2]. However, lack of a standardized approach can make treatment outcomes variable. We present the successful treatment of a GIH using preoperative progressive pneumoperitoneum (PPP) followed by simultaneous laparotomy hernia repair and transversus abdominis release (TAR).

**Figure 1:**
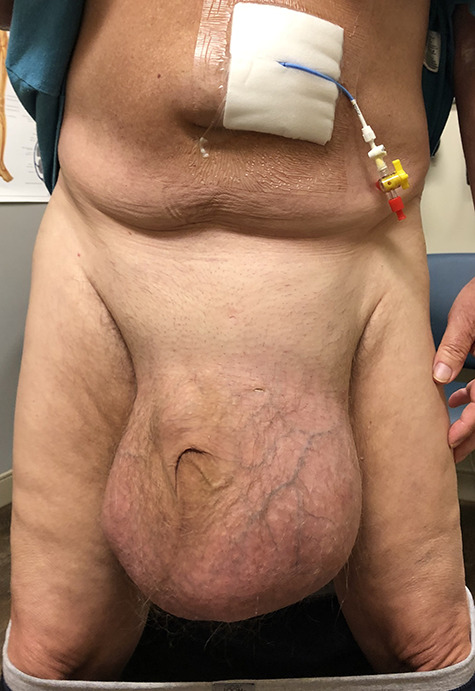
GIH extending past mid-thigh in upright position. Peritoneal port also shown above the umbilicus.

## CASE REPORT

**Figure 2:**
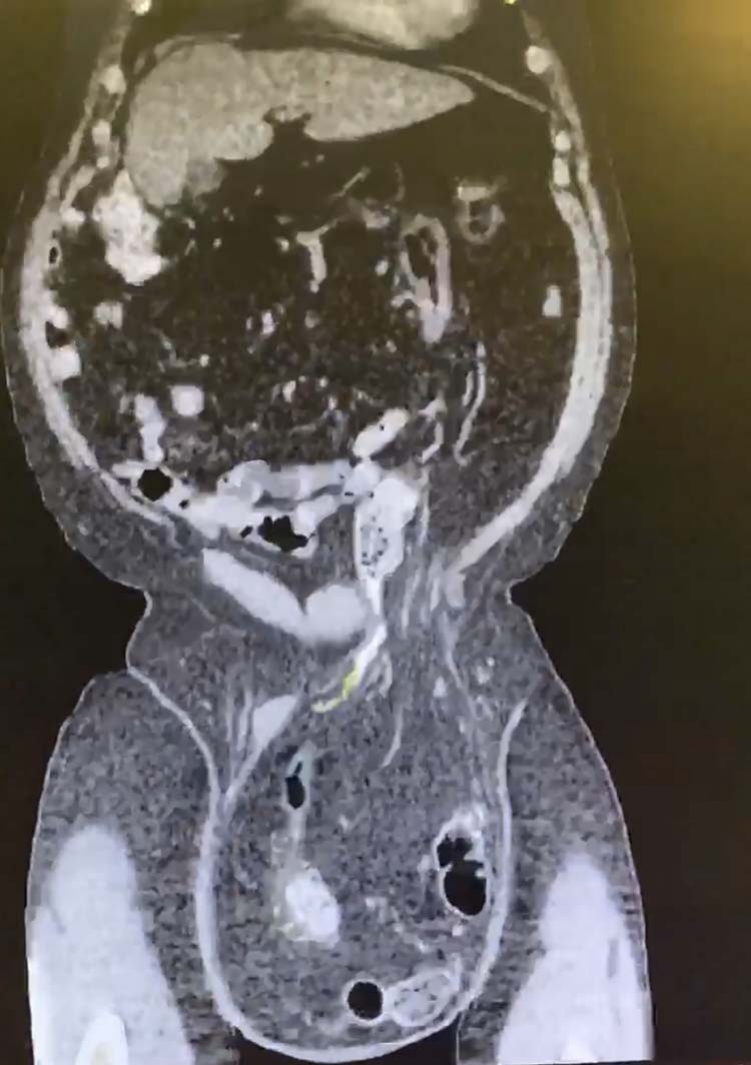
Coronal CT displaying sigmoid colon and accompanying abdominal contents traversing into the hernia sac.

**Figure 3:**
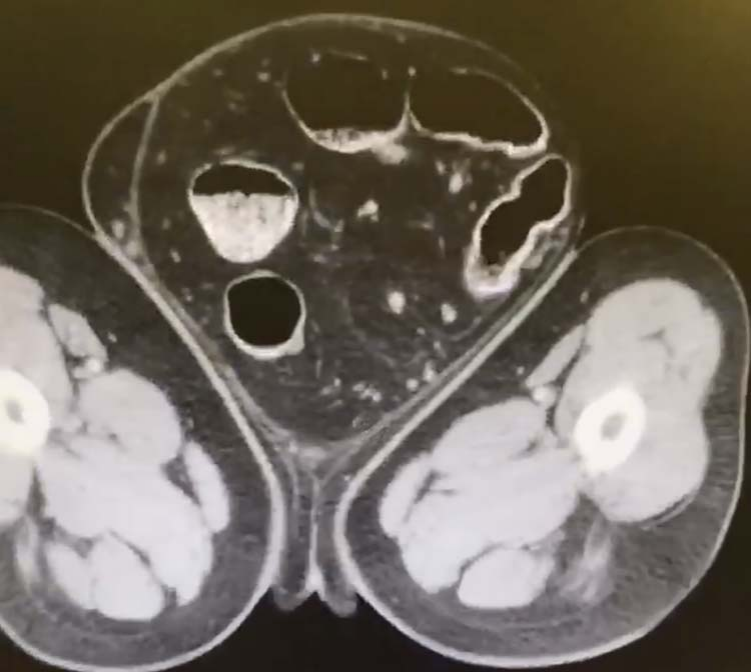
Transverse CT displaying colon within the hernia sac.

A 66-year-old male with a body mass index (BMI) of 36 presented to the clinic with a GIH of more than 10 years (Fig. 1). It has remained of similar size for the past 5 years and has caused discomfort but without frank pain. A chronically incarcerated umbilical hernia and small right inguinal hernia was also present on exam. Computed tomography (CT) scan showed large amounts of omentum, sigmoid colon, portions of transverse/descending colon and left ureter housed within the 5.7 L inguinoscrotal hernia sac (Figs 2 and 3). Additionally, the left ureter was tortuous and dilated, consequently causing moderate hydronephrosis. The patient was instructed to lose weight and undergo close follow-up in the outpatient setting for preoperative optimization of his preexisting medical conditions, hypertension and hypercholesteremia. He presented 9 months later with a BMI of 31, and an intra-peritoneal catheter was then placed to begin PPP. One liter of carbon dioxide per day was progressively insufflated for a total of 10 days. Concurrently, Deep vein thrombosis prophylaxis was initiated and continued throughout. Following pre-treatment of PPP, midline laparotomy hernia repair (Stoppa technique) was carried out. Manual reduction of incarcerated omentum and sigmoid colon were unsuccessful. Therefore, a counter longitudinal incision was made on the left scrotum, which allowed the sac to decompress for further ease of reduction. Moderate omentectomy was required to allow the delivery of residual omentum and sigmoid colon back into the abdominal cavity. Additionally, left orchiectomy was performed, given the high risk of potential torsion due to the lengthy and redundant pedicle residing in the new vacant hemiscrotum. With the contents fully reduced, a high amount of fascial tension remained. Thus, a bilateral TAR was proceeded in order to further assist in achieving a tension-free closure. Division of the transversus abdominis muscle fibers was attained by hydrodissection. Blunt dissection was then continued throughout the space of Bogros and the space of Retzius, both inferiorly and laterally. This allowed for the fascial layers to meet in the midline in a tension-free manner. Additionally, the small 3-cm right indirect inguinal hernia was identified and was sequentially reduced with an excision of a large cord lipoma. The hernia defects were closed and two 30 × 15 cm pro-grip meshes were placed in a preperitoneal fashion, laying over the myopectineal orifices bilaterally (Stoppa technique). The patient had an additional umbilical defect which was reduced and repaired using an additional piece of 30 × 15 cm pro-grip mesh, oriented cephalocaudally and placed in the retrorectus space for additional ventral support. The scrotal skin was left intact to allow for possible future scrotal reconstruction. The patient was extubated without respiratory complications and discharged home comfortably after an uncomplicated 7-day hospital stay. The patient required left scrotal seroma drainage 2 months postop, which consequently required the excision of the multiloculated cystic collection in the following month. Follow-up ensued once every 2 weeks to ensure optimal wound healing.

## DISCUSSION

The complexity behind treating GIHs persists due to the loss of abdominal volume, otherwise known as loss of domain. Techniques have been designed to combat the life-threatening complications of abdominal hypertension to either reduce the amount of contents delivered back into the abdomen (omentectomy, bowel resection) or to promote the regain of abdominal domain (PPP, TAR and botulinum toxin) [2, 5, 6]. PPP, first introduced by Moreno in 1947, has been used extensively in the management of large ventral incisional hernias (VIHs) and is regarded as a safe and beneficial preoperative measure for increasing the abdominal domain [7]. TAR is a newer technique which was first designed by Novitsky to overcome the obstacles encountered using the standard Rives–Stoppa technique when dealing with complex abdominal reconstruction [8]. The benefit of using a TAR approach allows for increased medial myofascial advancement while avoiding the disruption of the rectus abdominis neurovasculature and retaining native abdominal biomechanics. This convenient technique has applications across a variety of hernia types, however, to our knowledge has never been described for use in GIHs [9]. It is worth noting that a recent case describes the successful treatment of a GIH without the use of PPP, using an abdominal wall modified components separation technique instead, a method similar to what was performed in our patient [2]. Therefore, at what point is PPP warranted and at what volume remains to be subjective. Bueno-Lledó *et al.* [10] describe a promising algorithm for preoperative indications for PPP and botulinum toxin type A to treat large incisional hernias. The volume of VIH and the volume of abdominal cavity (VAC) were obtained via a CT scan and were used in a ratio to determine the need for preoperative measures. It was determined that if the ratio of VIH/VAC ≥ 20%, then preoperative measures were indicated for the reconstruction. This algorithm may clarify the necessity for pre-operative and intra-operative abdominal expansion measures in GIHs as well.

GIHs remain a complex entity, often requiring multi-stage procedures and/or careful preoperative planning. Here, we present a two-staged approach utilizing PPP, followed by the combined use of a hernia repair (Stoppa technique) with TAR to successfully treat a GIH with loss of domain.

## CONFLICT OF INTEREST STATEMENT

None declared.

## FUNDING

None.
